# Supplementary material for: Mapping Evidence on Management of Cervical Cancer in Sub-Saharan Africa: Scoping Review
Source: Int J Environ Res Public Health. 2022 Jul 28;19(15):9207. doi: 10.3390/ijerph19159207 (PMC9367747; doi:10.3390/ijerph19159207)
Supplement: Supplementary file 1 [file ijerph-19-09207-s001.zip › File S1- Search Strategy.pdf]

Search Strategy

Keyword	Alternatives
Cervical Cancer (P)	"Uterine Cervical Neoplasm"[Mesh] OR Cervical Neoplasm, Uterine OR Cervical Neoplasm, Uterine OR Neoplasm*OR Uterine Cervical OR Uterine Cervical Neoplasm* OR Neoplasm*, Cervical OR Neoplasm, Cervix OR Cervix Neoplasm* OR Neoplasm*, Cervix OR Cancer of the Uterine Cervix OR Cancer of the Cervix OR Cervical Cancer OR Uterine Cervical Cancer* OR Cancer*, Uterine Cervical OR Cervical Cancer*, Uterine OR Cancer of Cervix OR Cervix Cancer OR Cancer*, Cervix
Cervical Cancer Management (I)	cervical cancer management OR cervical cancer control OR cervical cancer Screening OR "Early Detection of Cancer"[Mesh] OR Screening, Mass OR Screenings, Mass OR Screening* OR Cancer Screening OR Screening, Cancer OR, HPV testing OR testing for human papillomavirus OR visual inspection with acetic acid OR VIAC OR Pap test OR Pap smear OR liquid-based cytology OR conventional cytology OR cervical cancer surgery OR Cervical cancer treatment OR cervical cancer chemotherapy OR Cervical cancer radiotherapy OR cervical cancer diagnosis OR Early Diagnosis of Cancer OR cervical cancer prevention OR human papillomavirus
Use of Filtering<br>Sub Saharan Africa	"Africa South of the Sahara"[Mesh] OR Sub-Saharan Africa OR Sub-Saharan Africa OR Africa, Sub-Saharan and to include all countries which form part of sub- Saharan Africa OR Angola OR Benin OR Botswana OR Burkina Faso OR Burundi OR Cameroon OR Central African Republic OR Chad OR Congo OR Cote d'Ivoire OR Democratic Republic of Congo OR Eritrea OR Ethiopia OR Gabon OR Gambia OR Ghana OR Guinea OR Guinea-Bissau OR Kenya OR Lesotho OR Liberia OR Madagascar OR Malawi OR Mali OR Mauritania OR Mauritius OR Mozambique OR Namibia OR Niger OR Nigeria OR Rwanda OR Senegal OR Sierra Leone OR Somalia OR South Africa OR United Republic of Tanzania OR Togo OR Uganda OR Zambia OR Zimbabwe
