# Supplementary material for: Mapping Evidence on Management of Cervical Cancer in Sub-Saharan Africa: Scoping Review
Source: Int J Environ Res Public Health. 2022 Jul 28;19(15):9207. doi: 10.3390/ijerph19159207 (PMC9367747; doi:10.3390/ijerph19159207)
Supplement: Supplementary file 1 [file ijerph-19-09207-s001.zip › File S3- Quality Assessment Tool.pdf]

Checklist for quality assessment (Mixed Method Quality Appraisal Tool) (37)

Quality criterion	Well covered	Adequately addressed	Poorly addressed	Not addressed	Not reported	N/A	Comments
1. Are the objectives or hypotheses of the study stated?	X						
2. Is the target population defined?		X					
3. Is the sampling frame defined?		X					
4. Is the study population defined?	X						
5. Are the study setting (venues) and/or geographic location stated?	X						well covered included the overall description in relation to the province (study area)
6. Are the dates between which the study was conducted stated or implicit?	X						
7. Are the eligibility criteria stated?	X						
8. Are the issues of 'selection in' to the study mentioned?	X						
9. Are the numbers of participants justified?	X						
10. Was the number of participants at the beginning of the study stated?	X						
11. Were the methods of data collection stated?	X						
12. Was the reliability (repeatability) of measurement methods mentioned?	X						as Quality assurance of the vaccination process
13. Are the methods of follow-up given?		X					
14. Was the number of participants at each stage/wave specified?	X						
15. Were the reasons for loss to follow-up quantified?	X						
16. Was the missing of data items at each wave mentioned?		X					
17. Were missing data accounted for in the analyses?	X						
18. Was the impact of biases estimated qualitatevely or quantitatively?					X		
19. Was there any other Discussion of generalizability?	X						
20. Overall assessment of the study (good quality; fair quality; poor quality) and comments	Good quality- every aspect was addressed except impact of biases, Lacked Triangulation of data collection methods						

Author & date: Black, E.Richmond, R.2019

Quality criterion	Well covered	Adequately addressed	Poorly addressed	Not addressed	Not reported	N/A	Comments

1. Are the objectives or hypotheses of the study stated?	X						
2. Is the target population defined?	X						
3. Is the sampling frame defined?	X						
4. Is the study population defined?	X						
5. Are the study setting (venues) and/or geographic location stated?	X						
6. Are the dates between which the study was conducted stated or implicit?	X						
7. Are the eligibility criteria stated?	X						
8. Are the issues of 'selection in' to the study mentioned?	X						
9. Are the numbers of participants justified?	X						
10. Was the number of participants at the beginning of the study stated?	X						
11. Were the methods of data collection stated?	X						
12. Was the reliability (repeatability) of measurement methods mentioned?		X					
13. Are the methods of follow-up given?		X					
14. Was the number of participants at each stage/wave specified?		X					
15. Were the reasons for loss to follow-up quantified?		X					
16. Was the missing of data items at each wave mentioned?		X					
17. Were missing data accounted for in the analyses?				x			The study was retrospectives hence no ways of getting missing data
18. Was the impact of biases estimated quantitatively or quantitatively?					X		
19. Was there any other Discussion of generalizability?	X						
20. Overall assessment of the study (good quality; fair quality; poor quality) and comments	Good quality- all aspects were covered except the issue of missing data						

Delany-MoretlweEt al 2018

Quality criterion	Well covered	Adequately addressed	Poorly addressed	Not addressed	Not reported	N/A	Comments
1. Are the objectives or hypotheses of the study stated?	X						
2. Is the target population defined?	X						
3. Is the sampling frame defined?	X						
4. Is the study population defined?	X						
5. Are the study setting (venues) and/or geographic location stated?	X						
6. Are the dates between which the study was conducted stated or implicit?	X						
7. Are the eligibility criteria stated?		X					Purposeful sampling used to select sites for the direct observation of vaccination sessions.
8. Are the issues of 'selection in' to the study mentioned?		X					Limited sampling of key informants
9. Are the numbers of participants justified?		X					
10. Was the number of participants at the beginning of the study stated?		X					
11. Were the methods of data collection stated?	X						
12. Was the reliability (repeatability) of measurement methods mentioned?		X					
13. Are the methods of follow-up given?	X						
14. Was the number of participants at each stage/wave specified?	X						
15. Were the reasons for loss to follow-up quantified?	X						
16. Was the missing data items at each wave mentioned?	X						
17. Were missing data accounted for in the analyses?	X				X		
18. Was the impact of biases estimated quantitatively or quantitatively?							
19. Was there any other Discussion of generalizability?	X						
20. Overall assessment of the study (good quality; fair quality; poor quality) and comments	Good quality- one researcher was responsible for conducting the document review						

Mabeya Et al 2018

Quality criterion	Well covered	Adequately addressed	Poorly addressed	Not addressed	Not reported	N/A	Comments
1. Are the objectives or hypotheses of the study stated?	X						
2. Is the target population defined?	X						
3. Is the sampling frame defined?	X						
4. Is the study population defined?	X						
5. Are the study setting (venues) and/or geographic location stated?	X						
6. Are the dates between which the study was conducted stated or implicit?	X						
7. Are the eligibility criteria stated?	X						
8. Are the issues of 'selection in' to the study mentioned?	X						
9. Are the numbers of participants justified?	X						
10. Was the number of participants at the beginning of the study stated?	X						
11. Were the methods of data collection stated?	X						
12. Was the reliability (repeatability) of measurement methods mentioned?	X						
13. Are the methods of follow-up given?	X						
14. Was the number of participants at each stage/wave specified?	X						
15. Were the reasons for loss to follow-up quantified?	X						
16. Was the missing data items at each wave mentioned?	X						
17. Were missing data accounted for in the analyses?	X						
18. Was the impact of biases estimated quantitatively or quantitatively?					X		lack of data on potential known confounders, including meteorological conditions
19. Was there any other Discussion of generalizability?	X						
20. Overall assessment of the study (good quality; fair quality; poor quality) and comments	Good quality- potential unknown confounders which may not have been identified						

Msyamboza Et al 2017

Quality criterion	Well covered	Adequately addressed	Poorly addressed	Not addressed	Not reported	N/A	Comments
1. Are the objectives or hypotheses of the study stated?	X						
2. Is the target population defined?	X						
3. Is the sampling frame defined?	X						
4. Is the study population defined?	X						
5. Are the study setting (venues) and/or geographic location stated?	X						
6. Are the dates between which the study was conducted stated or implicit?	X						
7. Are the eligibility criteria stated?	X						
8. Are the issues of 'selection in' to the study mentioned?	X						
9. Are the numbers of participants justified?	X						
10. Was the number of participants at the beginning of the study stated?	X						
11. Were the methods of data collection stated?	X						
12. Was the reliability (repeatability) of measurement methods mentioned?	X						
13. Are the methods of follow-up given?	X						
14. Was the number of participants at each stage/wave specified?	X						
15. Were the reasons for loss to follow-up quantified?	X						
16. Was the missing data items at each wave mentioned?	X						
17. Were missing data accounted for in the analyses?		X					no ways of getting missing data
18. Was the impact of biases estimated quantitatively or quantitatively?					X		
19. Was there any other Discussion of generalizability?	X						
20. Overall assessment of the study (good quality; fair quality; poor quality) and comments	Good quality- Recalling effect						

Ogembo Et al 2014

Quality criterion	Well covered	Adequately addressed	Poorly addressed	Not addressed	Not reported	N/A	Comments
1. Are the objectives or hypotheses of the study stated?	X						
2. Is the target population defined?	X						
3. Is the sampling frame defined?	X						
4. Is the study population defined?	X						
5. Are the study setting (venues) and/or geographic location stated?	X						
6. Are the dates between which the study was conducted stated or implicit?	X						
7. Are the eligibility criteria stated?	X						
8. Are the issues of 'selection in' to the study mentioned?	X						
9. Are the numbers of participants justified?	X						
10. Was the number of participants at the beginning of the study stated?	X						
11. Were the methods of data collection stated?	X						
12. Was the reliability (repeatability) of measurement methods mentioned?	X						
13. Are the methods of follow-up given?	X						The lack of knowledge of location of girls not in school.
14. Was the number of participants at each stage/wave specified?	X						
15. Were the reasons for loss to follow-up quantified?	X						
16. Was the missing data items at each wave mentioned?	X						
17. Were missing data accounted for in the analyses?	X						
18. Was the impact of biases estimated quantitatively or quantitatively?		X					
19. Was there any other Discussion of generalizability?	X						out-of-school girls required a different community outreach method
20. Overall assessment of the study (good quality; fair quality; poor quality) and comments	Good quality- Web-based data entry would have made use of the database more efficient						

Soi Et al 2018

Quality criterion	Well covered	Adequately addressed	Poorly addressed	Not addressed	Not reported	N/A	Comments
1. Are the objectives or hypotheses of the study stated?	X						
2. Is the target population defined?	X						
3. Is the sampling frame defined?	X						
4. Is the study population defined?	X						
5. Are the study setting (venues) and/or geographic location stated?	X						
6. Are the dates between which the study was conducted stated or implicit?	X						
7. Are the eligibility criteria stated?	X						
8. Are the issues of 'selection in' to the study mentioned?	X						
9. Are the numbers of participants justified?	X						
10. Was the number of participants at the beginning of the study stated?	X						
11. Were the methods of data collection stated?	X						
12. Was the reliability (repeatability) of measurement methods mentioned?	X						
13. Are the methods of follow-up given?		X					reaching out-of-school girls is a significant challenge
14. Was the number of participants at each stage/wave specified?	X						
15. Were the reasons for loss to follow-up quantified?	X						
16. Was the missing data items at each wave mentioned?		X					There were no ways of getting missing data
17. Were missing data accounted for in the analyses?	X						
18. Was the impact of biases estimated quantitatively or quantitatively?	X						Social desirability bias.
19. Was there any other Discussion of generalizability?		X					the representativeness of three districts in a country of 150 districts may be limited
20. Overall assessment of the study (good quality; fair quality; poor quality) and comments	Fair quality- participants were not interviewed(girls who were vaccinated)						

Vermandere Et al 2014

Quality criterion	Well covered	Adequately addressed	Poorly addressed	Not addressed	Not reported	N/A	Comments
1. Are the objectives or hypotheses of the study stated?	X						
2. Is the target population defined?	X						
3. Is the sampling frame defined?	X						
4. Is the study population defined?	X						
5. Are the study setting (venues) and/or geographic location stated?	X						
6. Are the dates between which the study was conducted stated or implicit?	X						
7. Are the eligibility criteria stated?	X						
8. Are the issues of 'selection in' to the study mentioned?	X						
9. Are the numbers of participants justified?	X						
10. Was the number of participants at the beginning of the study stated?	X						39% of the mothers invited at baseline did not participate
11. Were the methods of data collection stated?	X						
12. Was the reliability (repeatability) of measurement methods mentioned?	X						
13. Are the methods of follow-up given?	X						
14. Was the number of participants at each stage/wave specified?	X						
15. Were the reasons for loss to follow-up quantified?	X						absenteeism, drop-out, early sex and marriage, population mobility , distance from services and lack of parental support
16. Was the missing of data items at each wave mentioned?	X						
17. Were missing data accounted for in the analyses?	X						Missing registers, reluctance to disrupt classes to cross-check date of birth and inconsistent data.
18. Was the impact of biases estimated quantitatively or quantitatively?	X						
19. Was there any other discussion of generalizability?	X						
20. Overall assessment of the study (good quality; fair quality; poor quality) and comments	Good quality- social desirability						

Watson-Jones Et al 2012

Quality criterion	Well covered	Adequately addressed	Poorly addressed	Not addressed	Not reported	N/A	Comments
1. Are the objectives or hypotheses of the study stated?	X						
2. Is the target population defined?	X						
3. Is the sampling frame defined?	X						
4. Is the study population defined?	X						
5. Are the study setting (venues) and/or geographic location stated?	X						
6. Are the dates between which the study was conducted stated or implicit?	X						
7. Are the eligibility criteria stated?	X						
8. Are the issues of 'selection in' to the study mentioned?	X						Reluctance by some government schools to provide their records, incompleteness of data, missing attendance registers and unknown date of birth
9. Are the numbers of participants justified?	X						
10. Was the number of participants at the beginning of the study stated?	X						
11. Were the methods of data collection stated?	X						
12. Was the reliability (repeatability) of measurement methods mentioned?	X						
13. Are the methods of follow-up given?	X						
14. Was the number of participants at each stage/wave specified?	X						
15. Were the reasons for loss to follow-up quantified?	X						
16. Was the missing data items at each wave mentioned?	X						
17. Were missing data accounted for in the analyses?	X						
18. Was the impact of biases estimated quantitatively or quantitatively?					X		
19. Was there any other Discussion of generalizability?	X						
20. Overall assessment of the study (good quality; fair quality; poor quality) and comments	Good quality- School-based failed to reach 20% of unenrolled girls						

Quality criterion	Well covered	Adequately addressed	Poorly addressed	Not addressed	Not reported	N/A	Comments
1. Are the objectives or hypotheses of the study stated?	X						
2. Is the target population defined?	X						
3. Is the sampling frame defined?	X						
4. Is the study population defined?	X						
5. Are the study setting (venues) and/or geographic location stated?	X						
6. Are the dates between which the study was conducted stated or implicit?	X						
7. Are the eligibility criteria stated?	X						
8. Are the issues of 'selection in' to the study mentioned?	X						
9. Are the numbers of participants justified?	X						
10. Was the number of participants at the beginning of the study stated?	X						
11. Were the methods of data collection stated?	X						
12. Was the reliability (repeatability) of measurement methods mentioned?	X						
13. Are the methods of follow-up given?	X						
14. Was the number of participants at each stage/wave specified?	X						
15. Were the reasons for loss to follow-up quantified?	X						
16. Was the missing of data items at each wave mentioned?	X						
17. Were missing data accounted for in the analyses?	X						
18. Was the impact of biases estimated quantitatively or quantitatively?	X						Social desirability
19. Was there any other Discussion of generalizability?	X						
20. Overall assessment of the study (good quality; fair quality; poor quality) and comments	Good quality-Lacked triangulation of data collection methods						

Begoihn et al 2019

Quality criterion	Well covered	Adequately addressed	Poorly addressed	Not addressed	Not reported	N/A	Comments
1. Are the objectives or hypotheses of the study stated?	X						
2. Is the target population defined?	X						
3. Is the sampling frame defined?	X						
4. Is the study population defined?	X						
5. Are the study setting (venues) and/or geographic location stated?	X						
6. Are the dates between which the study was conducted stated or implicit?	X						
7. Are the eligibility criteria stated?	X						
8. Are the issues of 'selection in' to the study mentioned?	X						
9. Are the numbers of participants justified?	X						
10. Was the number of participants at the beginning of the study stated?	X						
11. Were the methods of data collection stated?	X						
12. Was the reliability (repeatability) of measurement methods mentioned?	X						
13. Are the methods of follow-up given?	X						
14. Was the number of participants at each stage/wave specified?	X						
15. Were the reasons for loss to follow-up quantified?	X						
16. Was the missing data items at each wave mentioned?	X						
17. Were missing data accounted for in the analyses?	X						
18. Was the impact of biases estimated quantitatively or quantitatively?		X					dates relied on self-reporting from patients who might have been subjected to recall bias
19. Was there any other Discussion of generalizability?	X						
20. Overall assessment of the study (good quality; fair quality; poor quality) and comments	Good quality- Data regarding patient and tumor characteristics and dates used for the calculation of patient interval were extracted from handwritten medical records.						

Dunyo, P. Effah, K. dofia, E. A. 2018

Quality criterion	Well covered	Adequately addressed	Poorly addressed	Not addressed	Not reported	N/A	Comments
1. Are the objectives or hypotheses of the study stated?	X						
2. Is the target population defined?	X						
3. Is the sampling frame defined?	X						
4. Is the study population defined?	X						
5. Are the study setting (venues) and/or geographic location stated?	X						
6. Are the dates between which the study was conducted stated or implicit?	X						
7. Are the eligibility criteria stated?	X						
8. Are the issues of 'selection in' to the study mentioned?	X						
9. Are the numbers of participants justified?	X						
10. Was the number of participants at the beginning of the study stated?	X						
11. Were the methods of data collection stated?	X						
12. Was the reliability (repeatability) of measurement methods mentioned?	X						
13. Are the methods of follow-up given?	X						
14. Was the number of participants at each stage/wave specified?	X						
15. Were the reasons for loss to follow-up quantified?	X						
16. Was the missing of data items at each wave mentioned?		X					The histopathology results often did not report on the tumor sub-type and characteristics of tumor.
17. Were missing data accounted for in the analyses?	X						
18. Was the impact of biases estimated quantitatively or quantitatively?	X						
19. Was there any other Discussion of generalizability?	X						
20. Overall assessment of the study (good quality; fair quality; poor quality) and comments	Good quality- sample size was small though justification was given						

Mlange Et al 2016

Quality criterion	Well covered	Adequately addressed	Poorly addressed	Not addressed	Not reported	N/A	Comments
1. Are the objectives or hypotheses of the study stated?	X						
2. Is the target population defined?	X						
3. Is the sampling frame defined?	X						
4. Is the study population defined?	X						
5. Are the study setting (venues) and/or geographic location stated?	X						
6. Are the dates between which the study was conducted stated or implicit?	X						
7. Are the eligibility criteria stated?	X						
8. Are the issues of 'selection in' to the study mentioned?	X						
9. Are the numbers of participants justified?	X						
10. Was the number of participants at the beginning of the study Stated?	X						
11. Were the methods of data collection stated?	X						
12. Was the reliability (repeatability) of measurement methods mentioned?	X						
13. Are the methods of follow-up given?	X						
14. Was the number of participants at each stage/wave specified?	X						
15. Were the reasons for loss to follow-up quantified?						N/A	
16. Was the missing of data items at each wave mentioned?	X						Investigations such as intravenous pyelogram (IVP), magnetic Resonance imaging (MRI), cystoscopy and urine for culture and sensitivity were not performed due to limited funds.
17. Were missing data accounted for in the analyses?	X						
18. Was the impact of biases estimated quantitatively or quantitatively?	X						
19. Was there any other Discussion of generalizability?	X						
20. Overall assessment of the study (good quality; fair quality; poor quality) and comments	Good quality-The study was quantitative only						

Mushosho Et al 2011

Quality criterion	Well covered	Adequately addressed	Poorly addressed	Not addressed	Not reported	N/A	Comments
1. Are the objectives or hypotheses of the study stated?	X						
2. Is the target population defined?	X						
3. Is the sampling frame defined?	X						
4. Is the study population defined?	X						
5. Are the study setting (venues) and/or geographic location stated?	X						
6. Are the dates between which the study was conducted stated or implicit?	X						
7. Are the eligibility criteria stated?	X						
8. Are the issues of 'selection in' to the study mentioned?	X						
9. Are the numbers of participants justified?	X						
10. Was the number of participants at the beginning of the study stated?	X						
11. Were the methods of data collection stated?	X						
12. Was the reliability (repeatability) of measurement methods mentioned?	X						
13. Are the methods of follow-up given?					X		
14. Was the number of participants at each stage/wave specified?	X						
15. Were the reasons for loss to follow-up quantified?					X		
16. Was the missing of data items at each wave mentioned?	X						There was no way of getting missing data since it was a retrospective study
17. Were missing data accounted for in the analyses?	X						
18. Was the impact of biases estimated quantitatively or quantitatively?	X						
19. Was there any other Discussion of generalizability?	X						
20. Overall assessment of the study (good quality; fair quality; poor quality) and comments	Good quality- no way of getting missing data in a retrospective study						

Mwaka et al 2016

Quality criterion	Well covered	Adequately addressed	Poorly addressed	Not addressed	Not reported	N/A	Comments
1. Are the objectives or hypotheses of the study stated?	X						
2. Is the target population defined?	X						
3. Is the sampling frame defined?	X						
4. Is the study population defined?	X						
5. Are the study setting (venues) and/or geographic location stated?	X						
6. Are the dates between which the study was conducted stated or implicit?	X						
7. Are the eligibility criteria stated?	X						
8. Are the issues of 'selection in' to the study mentioned?	X						
9. Are the numbers of participants justified?		X					
10. Was the number of participants at the beginning of the study stated?	X						
11. Were the methods of data collection stated?	X						
12. Was the reliability (repeatability) of measurement methods mentioned?	X						
13. Are the methods of follow-up given?						X	Cross -sectional study
14. Was the number of participants at each stage/wave specified?						X	
15. Were the reasons for loss to follow-up quantified?						X	
16. Was the missing data items at each wave mentioned?						X	
17. Were missing data accounted for in the analyses?	X						
18. Was the impact of biases estimated quantitatively or quantitatively?	X						Social desirebility
19. Was there any other Discussion of generalizability?		X					Hospital based
20. Overall assessment of the study (good quality; fair quality; poor quality) and comments	Good quality- affected by social desirability and recall bias						

Nassali Et al 2018

Quality criterion	Well covered	Adequately addressed	Poorly addressed	Not addressed	Not reported	N/A	Comments
1. Are the objectives or hypotheses of the study stated?	X						
2. Is the target population defined?	X						
3. Is the sampling frame defined?	X						
4. Is the study population defined?	X						
5. Are the study setting (venues) and/or geographic location stated?	X						
6. Are the dates between which the study was conducted stated or implicit?	X						
7. Are the eligibility criteria stated?	X						
8. Are the issues of 'selection in' to the study mentioned?	X						
9. Are the numbers of participants justified?	X						
10. Was the number of participants at the beginning of the study stated?	X						
11. Were the methods of data collection stated?	X						
12. Was the reliability (repeatability) of measurement methods mentioned?	X						
13. Are the methods of follow-up given?						X	
14. Was the number of participants at each stage/wave specified?						X	
15. Were the reasons for loss to follow-up quantified?						X	
16. Was the missing data items at each wave mentioned?	X						There was no way of getting missing data since the study was a retrospective study
17. Were missing data accounted for in the analyses?	X						
18. Was the impact of biases estimated quantitatively or quantitatively?	X						
19. Was there any other Discussion of generalizability?	X						
20. Overall assessment of the study (good quality; fair quality; poor quality) and comments	Good quality- not able to explore patient perspectives about their stage of presentation because this was a retrospective medical record Review						

Ndhlovu,N.Kambarami, R. 2003

Quality criterion	Well covered	Adequately addressed	Poorly addressed	Not addressed	Not reported	N/A	Comments
1. Are the objectives or hypotheses of the study stated?	X						
2. Is the target population defined?	X						
3. Is the sampling frame defined?		X					The sample size was small since
4. Is the study population defined?	X						
5. Are the study setting (venues) and/or geographic location stated?	X						
6. Are the dates between which the study was conducted stated or implicit?	X						
7. Are the eligibility criteria stated?	X						
8. Are the issues of 'selection in' to the study mentioned?	X						
9. Are the numbers of participants justified?		X					
10. Was the number of participants at the beginning of the study stated?	X						
11. Were the methods of data collection stated?	X						
12. Was the reliability (repeatability) of measurement methods mentioned?	X						
13. Are the methods of follow-up given?						X	
14. Was the number of participants at each stage/wave specified?						X	
15. Were the reasons for loss to follow-up quantified?						X	
16. Was the missing data items at each wave mentioned?	X						
17. Were missing data accounted for in the analyses?	X						
18. Was the impact of biases estimated quantitatively or quantitatively?	X						
19. Was there any other Discussion of generalizability?	X						
20. Overall assessment of the study (good quality; fair quality; poor quality) and comments	Good quality-but generalizability could be a problem because of the small sample size						

Rudd et al 2017

Quality criterion	Well covered	Adequately addressed	Poorly addressed	Not addressed	Not reported	N/A	Comments
1. Are the objectives or hypotheses of the study stated?	X						
2. Is the target population defined?	X						
3. Is the sampling frame defined?	X						
4. Is the study population defined?	X						
5. Are the study setting (venues) and/or geographic location stated?	X						
6. Are the dates between which the study was conducted stated or implicit?	X						
7. Are the eligibility criteria stated?	X						
8. Are the issues of 'selection in' to the study mentioned?	X						
9. Are the numbers of participants justified?	X						
10. Was the number of participants at the beginning of the study stated?	X						
11. Were the methods of data collection stated?	X						
12. Was the reliability (repeatability) of measurement methods mentioned?	X						
13. Are the methods of follow-up given?	X						
14. Was the number of participants at each stage/wave specified?	X						
15. Were the reasons for loss to follow-up quantified?	X						Follow up was poor
16. Was the missing data items at each wave mentioned?	X						
17. Were missing data accounted for in the analyses?	X						
18. Was the impact of biases estimated quantitatively or quantitatively?	X						
19. Was there any other Discussion of generalizability?	x						
20. Overall assessment of the study (good quality; fair quality; poor quality) and comments	Good quality- there was a some loss to follow up						

van Schalkwyk, S. L. Maree, J. E. Wright, S. C.2008

Quality criterion	Well covered	Adequately addressed	Poorly addressed	Not addressed	Not reported	N/A	Comments
1. Are the objectives or hypotheses of the study stated?	X						
2. Is the target population defined?	X						
3. Is the sampling frame defined?	X						
4. Is the study population defined?	X						
5. Are the study setting (venues) and/or geographic location stated?	X						
6. Are the dates between which the study was conducted stated or implicit?	X						
7. Are the eligibility criteria stated?	X						
8. Are the issues of 'selection in' to the study mentioned?	X						
9. Are the numbers of participants justified?	X						Sample size was small
10. Was the number of participants at the beginning of the study Stated?	X						
11. Were the methods of data collection stated?	X						
12. Was the reliability (repeatability) of measurement methods mentioned?	X						
13. Are the methods of follow-up given?						X	
14. Was the number of participants at each stage/wave specified?						X	
15. Were the reasons for loss to follow-up quantified?						X	
16. Was the missing data items at each wave mentioned?						X	
17. Were missing data accounted for in the analyses?						X	
18. Was the impact of biases estimated quantitatively or quantitatively?	X						
19. Was there any other Discussion of generalizability?	X						
20. Overall assessment of the study (good quality; fair quality; poor quality) and comments	Good quality-lack of triangulation of data collection method						

Wamburu Et al 2016

Quality criterion	Well covered	Adequately addressed	Poorly addressed	Not addressed	Not reported	N/A	Comments
1. Are the objectives or hypotheses of the study stated?	X						
2. Is the target population defined?	X						
3. Is the sampling frame defined?	X						
4. Is the study population defined?	X						
5. Are the study setting (venues) and/or geographic location stated?	X						
6. Are the dates between which the study was conducted stated or implicit?	X						
7. Are the eligibility criteria stated?	X						
8. Are the issues of 'selection in' to the study mentioned?	X						
9. Are the numbers of participants justified?	X						
10. Was the number of participants at the beginning of the study stated?	X						
11. Were the methods of data collection stated?	X						
12. Was the reliability (repeatability) of measurement methods mentioned?	X						
13. Are the methods of follow-up given?						X	
14. Was the number of participants at each stage/wave specified?						X	
15. Were the reasons for loss to follow-up quantified?						X	
16. Was the missing of data items at each wave mentioned?						X	
17. Were missing data accounted for in the analyses?	X						
18. Was the impact of biases estimated quantitatively or quantitatively?		X					Self reported data
19. Was there any other Discussion of generalizability?	X						
20. Overall assessment of the study (good quality; fair quality; poor quality) and comments	Good quality- social desirability is a limiting factor						

Bateman et al 2019

Quality criterion	Well covered	Adequately addressed	Poorly addressed	Not addressed	Not reported	N/A	Comments
1. Are the objectives or hypotheses of the study stated?	X						
2. Is the target population defined?	X						
3. Is the sampling frame defined?	X						
4. Is the study population defined?	X						
5. Are the study setting (venues) and/or geographic location stated?	X						
6. Are the dates between which the study was conducted stated or implicit?	X						
7. Are the eligibility criteria stated?	X						
8. Are the issues of 'selection in' to the study mentioned?	X						
9. Are the numbers of participants justified?		X					Small sample size
10. Was the number of participants at the beginning of the study stated?	X						
11. Were the methods of data collection stated?	X						
12. Was the reliability (repeatability) of measurement methods mentioned?	X						
13. Are the methods of follow-up given?						X	
14. Was the number of participants at each stage/wave specified?						X	
15. Were the reasons for loss to follow-up quantified?						X	
16. Was the missing of data items at each wave mentioned?						X	
17. Were missing data accounted for in the analyses?						X	
18. Was the impact of biases estimated quantitatively or quantitatively?	X						
19. Was there any other Discussion of generalizability?	X						
20. Overall assessment of the study (good quality; fair quality; poor quality) and comments	Good quality- narrow nature of the sample						

Binka, C. Nyarko, S. H.2019

Quality criterion	Well covered	Adequately addressed	Poorly addressed	Not addressed	Not reported	N/A	Comments
1. Are the objectives or hypotheses of the study stated?	X						
2. Is the target population defined?	X						
3. Is the sampling frame defined?	X						
4. Is the study population defined?	X						
5. Are the study setting (venues) and/or geographic location stated?	X						
6. Are the dates between which the study was conducted stated or implicit?	X						
7. Are the eligibility criteria stated?	X						
8. Are the issues of 'selection in' to the study mentioned?	X						
9. Are the numbers of participants justified?		X					Small sample size
10. Was the number of participants at the beginning of the study stated?	X						
11. Were the methods of data collection stated?	X						
12. Was the reliability (repeatability) of measurement methods mentioned?	X						
13. Are the methods of follow-up given?						X	
14. Was the number of participants at each stage/wave specified?						X	
15. Were the reasons for loss to follow-up quantified?						X	
16. Was the missing data items at each wave mentioned?						X	
17. Were missing data accounted for in the analyses?						X	
18. Was the impact of biases estimated quantitatively or quantitatively?	X						
19. Was there any other Discussion of generalizability?	X						
20. Overall assessment of the study (good quality; fair quality; poor quality) and comments	Good quality-lacked triangulation of data collection methods						

Bukirwa et al 2015

Quality criterion	Well covered	Adequately addressed	Poorly addressed	Not addressed	Not reported	N/A	Comments
1. Are the objectives or hypotheses of the study stated?	X						
2. Is the target population defined?	X						
3. Is the sampling frame defined?	X						
4. Is the study population defined?	X						
5. Are the study setting (venues) and/or geographic location stated?	X						
6. Are the dates between which the study was conducted stated or implicit?	X						
7. Are the eligibility criteria stated?	X						
8. Are the issues of 'selection in' to the study mentioned?	X						
9. Are the numbers of participants justified?		X					The sample size was small
10. Was the number of participants at the beginning of the study stated?	X						
11. Were the methods of data collection stated?	X						
12. Was the reliability (repeatability) of measurement methods mentioned?	X						
13. Are the methods of follow-up given?						X	
14. Was the number of participants at each stage/wave specified?						X	
15. Were the reasons for loss to follow-up quantified?						X	
16. Was the missingness of data items at each wave mentioned?						X	
17. Were missing data accounted for in the analyses?						X	
18. Was the impact of biases estimated quantitatively or quantitatively?	X						
19. Was there any other Discussion of generalizability?	X						
20. Overall assessment of the study (good quality; fair quality; poor quality) and comments	Good quality- lacked triangulation of data collection methods, sample size was small, need to further evaluate the findings from this qualitative study in a larger population-based survey						

Getachew, S. Getachew, E. 2019

Quality criterion	Well covered	Adequately addressed	Poorly addressed	Not addressed	Not reported	N/A	Comments
1. Are the objectives or hypotheses of the study stated?	X						
2. Is the target population defined?	X						
3. Is the sampling frame defined?	X						
4. Is the study population defined?	X						
5. Are the study setting (venues) and/or geographic location stated?	X						
6. Are the dates between which the study was conducted stated or implicit?	X						
7. Are the eligibility criteria stated?	X						
8. Are the issues of 'selection in' to the study mentioned?	X						
9. Are the numbers of participants justified?							
10. Was the number of participants at the beginning of the study stated?	X						
11. Were the methods of data collection stated?	X						
12. Was the reliability (repeatability) of measurement methods mentioned?	X						
13. Are the methods of follow-up given?						X	
14. Was the number of participants at each stage/wave specified?						X	
15. Were the reasons for loss to follow-up quantified?						X	
16. Was the missing of data items at each wave mentioned?						X	
17. Were missing data accounted for in the analyses?						X	
18. Was the impact of biases estimated quantitatively or quantitatively?		X					Social desirability
19. Was there any other Discussion of generalizability?	X						
20. Overall assessment of the study (good quality; fair quality; poor quality) and comments	Good quality- The study has some limitations in using the cross-sectional study design which might have a causative effect between determining factors and knowledge about CC screening						

Ilevbare, O. E.Adegoke, A. A.Adelowo, C. M. 2020

Quality criterion	Well covered	Adequately addressed	Poorly addressed	Not addressed	Not reported	N/A	Comments
1. Are the objectives or hypotheses of the study stated?	X						
2. Is the target population defined?	X						
3. Is the sampling frame defined?	X						
4. Is the study population defined?	X						
5. Are the study setting (venues) and/or geographic location stated?	X						
6. Are the dates between which the study was conducted stated or implicit?	X						
7. Are the eligibility criteria stated?	X						
8. Are the issues of 'selection in' to the study mentioned?	X						
9. Are the numbers of participants justified?	X						
10. Was the number of participants at the beginning of the study stated?	X						
11. Were the methods of data collection stated?	X						
12. Was the reliability (repeatability) of measurement methods mentioned?	X						
13. Are the methods of follow-up given?						X	
14. Was the number of participants at each stage/wave specified?						X	
15. Were the reasons for loss to follow-up quantified?						X	
16. Was the missing of data items at each wave mentioned?						X	
17. Were missing data accounted for in the analyses?						X	
18. Was the impact of biases estimated quantitatively or quantitatively?		X					Social desirability
19. Was there any other Discussion of generalizability?	x						
20. Overall assessment of the study (good quality; fair quality; poor quality) and comments	Good quality-lacked triangulation of data collection methods, need to further evaluate the findings from this study in a qualitative study						

Isa Modibbo, F.Dareng, E.Bamisaye, P. 2016

Quality criterion	Well covered	Adequately addressed	Poorly addressed	Not addressed	Not reported	N/A	Comments
1. Are the objectives or hypotheses of the study stated?	X						
2. Is the target population defined?	X						
3. Is the sampling frame defined?	X						
4. Is the study population defined?	X						
5. Are the study setting (venues) and/or geographic location stated?	X						
6. Are the dates between which the study was conducted stated or implicit?	X						
7. Are the eligibility criteria stated?	X						
8. Are the issues of 'selection in' to the study mentioned?	X						
9. Are the numbers of participants justified?		X					Small sample size
10. Was the number of participants at the beginning of the study stated?	X						
11. Were the methods of data collection stated?	X						
12. Was the reliability (repeatability) of measurement methods mentioned?	X						
13. Are the methods of follow-up given?						X	
14. Was the number of participants at each stage/wave specified?						X	
15. Were the reasons for loss to follow-up quantified?						X	
16. Was the missing of data items at each wave mentioned?						X	
17. Were missing data accounted for in the analyses?						X	
18. Was the impact of biases estimated quantitatively or quantitatively?	X						
19. Was there any other discussion of generalizability?	X						
20. Overall assessment of the study (good quality; fair quality; poor quality) and comments	Good quality-lacked triangulation of data collection methods						

Kivuti-Bitok et al 2013

Quality criterion	Well covered	Adequately addressed	Poorly addressed	Not addressed	Not reported	N/A	Comments
1. Are the objectives or hypotheses of the study stated?	X						
2. Is the target population defined?	X						
3. Is the sampling frame defined?	X						
4. Is the study population defined?	X						
5. Are the study setting (venues) and/or geographic location stated?	X						
6. Are the dates between which the study was conducted stated or implicit?	X						
7. Are the eligibility criteria stated?	X						
8. Are the issues of 'selection in' to the study mentioned?	X						
9. Are the numbers of participants justified?	X						
10. Was the number of participants at the beginning of the study stated?	X						
11. Were the methods of data collection stated?	X						
12. Was the reliability (repeatability) of measurement methods mentioned?	X						
13. Are the methods of follow-up given?						X	
14. Was the number of participants at each stage/wave specified?						X	
15. Were the reasons for loss to follow-up quantified?						X	
16. Was the missing data items at each wave mentioned?						X	
17. Were missing data accounted for in the analyses?						X	
18. Was the impact of biases estimated quantitatively or quantitatively?	X						Narrowed perspective
19. Was there any other Discussion of generalizability?		X					
20. Overall assessment of the study (good quality; fair quality; poor quality) and comments	Good quality- lacked patients' point of view						

Lee Et al 2020

Quality criterion	Well covered	Adequately addressed	Poorly addressed	Not addressed	Not reported	N/A	Comments
1. Are the objectives or hypotheses of the study stated?	X						
2. Is the target population defined?	X						
3. Is the sampling frame defined?	X						
4. Is the study population defined?	X						
5. Are the study setting (venues) and/or geographic location stated?	X						
6. Are the dates between which the study was conducted stated or implicit?	X						
7. Are the eligibility criteria stated?	X						
8. Are the issues of 'selection in' to the study mentioned?	X						
9. Are the numbers of participants justified?		X					Small sample size
10. Was the number of participants at the beginning of the study stated?	X						
11. Were the methods of data collection stated?	X						
12. Was the reliability (repeatability) of measurement methods mentioned?	X						
13. Are the methods of follow-up given?						X	
14. Was the number of participants at each stage/wave specified?						X	
15. Were the reasons for loss to follow-up quantified?						X	
16. Was the missing of data items at each wave mentioned?						X	
17. Were missing data accounted for in the analyses?						X	
18. Was the impact of biases estimated quantitatively or quantitatively?	X						
19. Was there any other Discussion of generalizability?	X						
20. Overall assessment of the study (good quality; fair quality; poor quality) and comments	Good quality-lacked triangulation of data collection methods						

Maseko, F. C. Chirwa, M. L. Muula, A. S.2015

Quality criterion	Well covered	Adequately addressed	Poorly addressed	Not addressed	Not reported	N/A	Comments
1. Are the objectives or hypotheses of the study stated?	X						
2. Is the target population defined?	X						
3. Is the sampling frame defined?	X						
4. Is the study population defined?	X						
5. Are the study setting (venues) and/or geographic location stated?	X						
6. Are the dates between which the study was conducted stated or implicit?	X						
7. Are the eligibility criteria stated?	X						
8. Are the issues of 'selection in' to the study mentioned?	X						
9. Are the numbers of participants justified?		X					Small sample size
10. Was the number of participants at the beginning of the study stated?	X						
11. Were the methods of data collection stated?	X						
12. Was the reliability (repeatability) of measurement methods mentioned?	X						
13. Are the methods of follow-up given?						X	
14. Was the number of participants at each stage/wave specified?						X	
15. Were the reasons for loss to follow-up quantified?						X	
16. Was the missing data items at each wave mentioned?						X	
17. Were missing data accounted for in the analyses?						X	
18. Was the impact of biases estimated quantitatively or quantitatively?		X					Social desirebility
19. Was there any other Discussion of generalizability?	X						
20. Overall assessment of the study (good quality; fair quality; poor quality) and comments	Good quality- lacked triangulation of data collection methods						

Munthali, A. C. Ngwira, B. M.aulo, F. 2015

Quality criterion	Well covered	Adequately addressed	Poorly addressed	Not addressed	Not reported	N/A	Comments
1. Are the objectives or hypotheses of the study stated?	X						
2. Is the target population defined?	X						
3. Is the sampling frame defined?	X						
4. Is the study population defined?	X						
5. Are the study setting (venues) and/or geographic location stated?	X						
6. Are the dates between which the study was conducted stated or implicit?	X						
7. Are the eligibility criteria stated?	X						
8. Are the issues of 'selection in' to the study mentioned?	X						
9. Are the numbers of participants justified?	X						
10. Was the number of participants at the beginning of the study stated?	X						
11. Were the methods of data collection stated?	X						
12. Was the reliability (repeatability) of measurement methods mentioned?	X						
13. Are the methods of follow-up given?							
14. Was the number of participants at each stage/wave specified?						X	
15. Were the reasons for loss to follow-up quantified?						X	
16. Was the missingness of data items at each wave mentioned?						X	
17. Were missing data accounted for in the analyses?						X	
18. Was the impact of biases estimated quantitatively or quantitatively?		X					Social desirability
19. Was there any other Discussion of generalizability?	X						
20. Overall assessment of the study (good quality; fair quality; poor quality) and comments	Good quality- lacked triangulation of data collection methods						

Ndejjo Et al 2016

Quality criterion	Well covered	Adequately addressed	Poorly addressed	Not addressed	Not reported	N/A	Comments
1. Are the objectives or hypotheses of the study stated?	X						
2. Is the target population defined?	X						
3. Is the sampling frame defined?	X						
4. Is the study population defined?	X						
5. Are the study setting (venues) and/or geographic location stated?	X						
6. Are the dates between which the study was conducted stated or implicit?	X						
7. Are the eligibility criteria stated?	X						
8. Are the issues of 'selection in' to the study mentioned?	X						
9. Are the numbers of participants justified?	X						
10. Was the number of participants at the beginning of the study stated?						X	
11. Were the methods of data collection stated?	X						
12. Was the reliability (repeatability) of measurement methods mentioned?	X						
13. Are the methods of follow-up given?						X	
14. Was the number of participants at each stage/wave specified?						X	
15. Were the reasons for loss to follow-up quantified?						X	
16. Was the missing of data items at each wave mentioned?						X	
17. Were missing data accounted for in the analyses?						X	
18. Was the impact of biases estimated quantitatively or quantitatively?		X					Social desirability
19. Was there any other Discussion of generalizability?	X						
20. Overall assessment of the study (good quality; fair quality; poor quality) and comments	Good quality- being a cross sectional study, it is not possible to assess causality						

Shiferaw, S Addissie, A. 2018

Quality criterion	Well covered	Adequately addressed	Poorly addressed	Not addressed	Not reported	N/A	Comments
1. Are the objectives or hypotheses of the study stated?	X						
2. Is the target population defined?	X						
3. Is the sampling frame defined?	X						
4. Is the study population defined?	X						
5. Are the study setting (venues) and/or geographic location stated?	X						
6. Are the dates between which the study was conducted stated or implicit?	X						
7. Are the eligibility criteria stated?	X						
8. Are the issues of 'selection in' to the study mentioned?	X						
9. Are the numbers of participants justified?	X						
10. Was the number of participants at the beginning of the study stated?						X	
11. Were the methods of data collection stated?	X						
12. Was the reliability (repeatability) of measurement methods mentioned?	X						
13. Are the methods of follow-up given?						X	
14. Was the number of participants at each stage/wave specified?						X	
15. Were the reasons for loss to follow-up quantified?						X	
16. Was the missing of data items at each wave mentioned?						X	
17. Were missing data accounted for in the analyses?						X	
18. Was the impact of biases estimated quantitatively or quantitatively?		X					interviewer bias
19. Was there any other Discussion of generalizability?	X						
20. Overall assessment of the study (good quality; fair quality; poor quality) and comments	Good quality-social desirability, the study excludes HIV positive women who receive HIV care from private healthcare institutions						

Were, E. Nyaberi, Z. Buziba, N. 2011

Quality criterion	Well covered	Adequately addressed	Poorly addressed	Not addressed	Not reported	N/A	Comments
1. Are the objectives or hypotheses of the study stated?	X						
2. Is the target population defined?	X						
3. Is the sampling frame defined?	X						
4. Is the study population defined?	X						
5. Are the study setting (venues) and/or geographic location stated?	X						
6. Are the dates between which the study was conducted stated or implicit?	X						
7. Are the eligibility criteria stated?	X						
8. Are the issues of 'selection in' to the study mentioned?	X						
9. Are the numbers of participants justified?	X						
10. Was the number of participants at the beginning of the study stated?						X	
11. Were the methods of data collection stated?	X						
12. Was the reliability (repeatability) of measurement methods mentioned?	X						
13. Are the methods of follow-up given?						X	
14. Was the number of participants at each stage/wave specified?						X	
15. Were the reasons for loss to follow-up quantified?						X	
16. Was the missing data items at each wave mentioned?						X	
17. Were missing data accounted for in the analyses?						X	
18. Was the impact of biases estimated quantitatively or quantitatively?		X					Social desirability
19. Was there any other Discussion of generalizability?	X						
20. Overall assessment of the study (good quality; fair quality; poor quality) and comments	Good quality-interviewer bias, women who were already accessing the services and therefore not representative of women in the reproductive age group from the catchment						

Williams Et al 2013

Quality criterion	Well covered	Adequately addressed	Poorly addressed	Not addressed	Not reported	N/A	Comments
1. Are the objectives or hypotheses of the study stated?	X						
2. Is the target population defined?	X						
3. Is the sampling frame defined?	X						
4. Is the study population defined?	X						
5. Are the study setting (venues) and/or geographic location stated?	X						
6. Are the dates between which the study was conducted stated or implicit?	X						
7. Are the eligibility criteria stated?	X						
8. Are the issues of 'selection in' to the study mentioned?	X						
9. Are the numbers of participants justified?	X						
10. Was the number of participants at the beginning of the study stated?						X	
11. Were the methods of data collection stated?	X						
12. Was the reliability (repeatability) of measurement methods mentioned?	X						
13. Are the methods of follow-up given?						X	
14. Was the number of participants at each stage/wave specified?						X	
15. Were the reasons for loss to follow-up quantified?						X	
16. Was the missing data items at each wave mentioned?						X	
17. Were missing data accounted for in the analyses?						X	
18. Was the impact of biases estimated quantitatively or quantitatively?		X					Social desirability
19. Was there any other Discussion of generalizability?	X						
20. Overall assessment of the study (good quality; fair quality; poor quality) and comments	Good quality-the study implemented triangulation of data collection methods						

Einstein Et al 2019

Quality criterion	Well covered	Adequately addressed	Poorly addressed	Not addressed	Not reported	N/A	Comments
1. Are the objectives or hypotheses of the study stated?	X						
2. Is the target population defined?	X						
3. Is the sampling frame defined?	X						
4. Is the study population defined?	X						
5. Are the study setting (venues) and/or geographic location stated?	X						
6. Are the dates between which the study was conducted stated or implicit?	X						
7. Are the eligibility criteria stated?	X						
8. Are the issues of 'selection in' to the study mentioned?	X						
9. Are the numbers of participants justified?		X					Sample size small
10. Was the number of participants at the beginning of the study stated?	X						
11. Were the methods of data collection stated?	X						
12. Was the reliability (repeatability) of measurement methods mentioned?	X						
13. Are the methods of follow-up given?	X						
14. Was the number of participants at each stage/wave specified?	X						
15. Were the reasons for loss to follow-up quantified?	X						
16. Was the missing of data items at each wave mentioned?						X	
17. Were missing data accounted for in the analyses?						X	
18. Was the impact of biases estimated quantitatively or quantitatively?	X						
19. Was there any other Discussion of generalizability?		X					
20. Overall assessment of the study (good quality; fair quality; poor quality) and comments	Good quality- phase2 clinical trials						

Marteiet al 2018

Quality criterion	Well covered	Adequately addressed	Poorly addressed	Not addressed	Not reported	N/A	Comments
1. Are the objectives or hypotheses of the study stated?	X						
2. Is the target population defined?	X						
3. Is the sampling frame defined?	X						
4. Is the study population defined?	X						
5. Are the study setting (venues) and/or geographic location stated?	X						
6. Are the dates between which the study was conducted stated or implicit?	X						
7. Are the eligibility criteria stated?	X						
8. Are the issues of 'selection in' to the study mentioned?	X						
9. Are the numbers of participants justified?	X						
10. Was the number of participants at the beginning of the study stated?						X	
11. Were the methods of data collection stated?	X						
12. Was the reliability (repeatability) of measurement methods mentioned?	X						
13. Are the methods of follow-up given?						X	
14. Was the number of participants at each stage/wave specified?						X	
15. Were the reasons for loss to follow-up quantified?	X						
16. Was the missing data items at each wave mentioned?	X						
17. Were missing data accounted for in the analyses?	X						
18. Was the impact of biases estimated quantitatively or quantitatively?		X					Small sample size
19. Was there any other Discussion of generalizability?	X						
20. Overall assessment of the study (good quality; fair quality; poor quality) and comments	Good quality- Sample size of drugs purchased was small consequently may have missed cost associated with stock outs.						

McArdle, O. Kigula-Mugambe, J. B. 2007

Quality criterion	Well covered	Adequately addressed	Poorly addressed	Not addressed	Not reported	N/A	Comments
1. Are the objectives or hypotheses of the study stated?	X						
2. Is the target population defined?	X						
3. Is the sampling frame defined?	X						
4. Is the study population defined?	X						
5. Are the study setting (venues) and/or geographic location stated?	X						
6. Are the dates between which the study was conducted stated or implicit?	X						
7. Are the eligibility criteria stated?	X						
8. Are the issues of 'selection in' to the study mentioned?	X						
9. Are the numbers of participants justified?	X						
10. Was the number of participants at the beginning of the study stated?	X						
11. Were the methods of data collection stated?	X						
12. Was the reliability (repeatability) of measurement methods mentioned?	X						
13. Are the methods of follow-up given?	X						
14. Was the number of participants at each stage/wave specified?	X						
15. Were the reasons for loss to follow-up quantified?		X					Follow up was poor
16. Was the missing of data items at each wave mentioned?	X						
17. Were missing data accounted for in the analyses?	X						
18. Was the impact of biases estimated quantitatively or quantitatively?	X						
19. Was there any other Discussion of generalizability?	X						
20. Overall assessment of the study (good quality; fair quality; poor quality) and comments	good quality-data was captured manually						

Simonds Et al 2012

Quality criterion	Well covered	Adequately addressed	Poorly addressed	Not addressed	Not reported	N/A	Comments
1. Are the objectives or hypotheses of the study stated?	X						
2. Is the target population defined?	X						
3. Is the sampling frame defined?	X						
4. Is the study population defined?	X						
5. Are the study setting (venues) and/or geographic location stated?	X						
6. Are the dates between which the study was conducted stated or implicit?	X						
7. Are the eligibility criteria stated?	X						
8. Are the issues of 'selection in' to the study mentioned?	X						
9. Are the numbers of participants justified?		X					Small sample size
10. Was the number of participants at the beginning of the study stated?						X	
11. Were the methods of data collection stated?	X						
12. Was the reliability (repeatability) of measurement methods mentioned?	X						
13. Are the methods of follow-up given?						X	
14. Was the number of participants at each stage/wave specified?						X	
15. Were the reasons for loss to follow-up quantified?						X	
16. Was the missing of data items at each wave mentioned?	X						
17. Were missing data accounted for in the analyses?		X					No way of getting missing data in a retrospective study
18. Was the impact of biases estimated quantitatively or quantitatively?	X						
19. Was there any other Discussion of generalizability?	X						
20. Overall assessment of the study (good quality; fair quality; poor quality) and comments	Fair quality- HIV-positive sample underpowered to detect differences in some characteristics and outcomes.						

Akinlade et al 2015

Quality criterion	Well covered	Adequately addressed	Poorly addressed	Not addressed	Not reported	N/A	Comments
1. Are the objectives or hypotheses of the study stated?	X						
2. Is the target population defined?	X						
3. Is the sampling frame defined?	X						
4. Is the study population defined?	X						
5. Are the study setting (venues) and/or geographic location stated?	X						
6. Are the dates between which the study was conducted stated or implicit?	X						
7. Are the eligibility criteria stated?	X						
8. Are the issues of 'selection in' to the study mentioned?	X						
9. Are the numbers of participants justified?	X						
10. Was the number of participants at the beginning of the study stated?						X	
11. Were the methods of data collection stated?	X						
12. Was the reliability (repeatability) of measurement methods mentioned?	X						
13. Are the methods of follow-up given?						X	
14. Was the number of participants at each stage/wave specified?						X	
15. Were the reasons for loss to follow-up quantified?		X					
16. Was the missing data items at each wave mentioned?	X						
17. Were missing data accounted for in the analyses?		X					Retrospective study
18. Was the impact of biases estimated quantitatively or quantitatively?	X						
19. Was there any other Discussion of generalizability?	X						
20. Overall assessment of the study (good quality; fair quality; poor quality) and comments	Good quality- insufficient follow-up of patients						

Du Toit, G. C. Kidd, M.2015

Quality criterion	Well covered	Adequately addressed	Poorly addressed	Not addressed	Not reported	N/A	Comments
1. Are the objectives or hypotheses of the study stated?	X						
2. Is the target population defined?	X						
3. Is the sampling frame defined?	X						
4. Is the study population defined?	X						
5. Are the study setting (venues) and/or geographic location stated?	X						
6. Are the dates between which the study was conducted stated or implicit?	X						
7. Are the eligibility criteria stated?	X						
8. Are the issues of 'selection in' to the study mentioned?	X						
9. Are the numbers of participants justified?	X						
10. Was the number of participants at the beginning of the study stated?	X						
11. Were the methods of data collection stated?	X						
12. Was the reliability (repeatability) of measurement methods mentioned?	X						
13. Are the methods of follow-up given?	X						
14. Was the number of participants at each stage/wave specified?	X						
15. Were the reasons for loss to follow-up quantified?		X					Follow up was poor
16. Was the missing data items at each wave mentioned?	X						
17. Were missing data accounted for in the analyses?	X						
18. Was the impact of biases estimated quantitatively or quantitatively?	X						
19. Was there any other Discussion of generalizability?	X						
20. Overall assessment of the study (good quality; fair quality; poor quality) and comments	Good quality- small numbers that preclude quality of life analysis of subgroups						

Einck et al 2014

Quality criterion	Well covered	Adequately addressed	Poorly addressed	Not addressed	Not reported	N/A	Comments
1. Are the objectives or hypotheses of the study stated?	X						
2. Is the target population defined?	X						
3. Is the sampling frame defined?	X						
4. Is the study population defined?	X						
5. Are the study setting (venues) and/or geographic location stated?	X						
6. Are the dates between which the study was conducted stated or implicit?	X						
7. Are the eligibility criteria stated?	X						
8. Are the issues of 'selection in' to the study mentioned?	X						
9. Are the numbers of participants justified?	X						
10. Was the number of participants at the beginning of the study stated?						X	
11. Were the methods of data collection stated?	X						
12. Was the reliability (repeatability) of measurement methods mentioned?	X						
13. Are the methods of follow-up given?		X					Follow up was poor
14. Was the number of participants at each stage/wave specified?						X	
15. Were the reasons for loss to follow-up quantified?	X					X	
16. Was the missing data items at each wave mentioned?	X						
17. Were missing data accounted for in the analyses?	X						
18. Was the impact of biases estimated quantitatively or quantitatively?	X						
19. Was there any other Discussion of generalizability?	X						
20. Overall assessment of the study (good quality; fair quality; poor quality) and comments	Good quality- No proper assessment of treated patients was fully done and documented						

Kamau, R. K. Osoti, A. O. Njuguna, E. M.2007

Quality criterion	Well covered	Adequately addressed	Poorly addressed	Not addressed	Not reported	N/A	Comments
1. Are the objectives or hypotheses of the study stated?	X						
2. Is the target population defined?	X						
3. Is the sampling frame defined?	X						
4. Is the study population defined?	X						
5. Are the study setting (venues) and/or geographic location stated?	X						
6. Are the dates between which the study was conducted stated or implicit?	X						
7. Are the eligibility criteria stated?	X						
8. Are the issues of 'selection in' to the study mentioned?	X						
9. Are the numbers of participants justified?		X					Small sample size
10. Was the number of participants at the beginning of the study stated?						X	
11. Were the methods of data collection stated?	X						
12. Was the reliability (repeatability) of measurement methods mentioned?	X						
13. Are the methods of follow-up given?						X	
14. Was the number of participants at each stage/wave specified?						X	
15. Were the reasons for loss to follow-up quantified?							
16. Was the missing data items at each wave mentioned?						X	
17. Were missing data accounted for in the analyses?						X	
18. Was the impact of biases estimated quantitatively or quantitatively?		X					
19. Was there any other Discussion of generalizability?	X						
20. Overall assessment of the study (good quality; fair quality; poor quality) and comments	Good quality-social desirability						

Kantelhardt Et al 2014

Quality criterion	Well covered	Adequately addressed	Poorly addressed	Not addressed	Not reported	N/A	Comments
1. Are the objectives or hypotheses of the study stated?	X						
2. Is the target population defined?	X						
3. Is the sampling frame defined?	X						
4. Is the study population defined?	X						
5. Are the study setting (venues) and/or geographic location stated?	X						
6. Are the dates between which the study was conducted stated or implicit?	X						
7. Are the eligibility criteria stated?	X						
8. Are the issues of 'selection in' to the study mentioned?	X						
9. Are the numbers of participants justified?	X						
10. Was the number of participants at the beginning of the study stated?	X						
11. Were the methods of data collection stated?	X						
12. Was the reliability (repeatability) of measurement methods mentioned?	X						
13. Are the methods of follow-up given?							
14. Was the number of participants at each stage/wave specified?		X					lacks late-stage patients who died before start of therapy
15. Were the reasons for loss to follow-up quantified?		X					
16. Was the missing data items at each wave mentioned?		X					unable to retrieve almost 400 files (27%) belonging to patients who received therapy
17. Were missing data accounted for in the analyses?		X					
18. Was the impact of biases estimated quantitatively or quantitatively?		X					poor patients unable to pay for therapy were excluded
19. Was there any other Discussion of generalizability?	X						
20. Overall assessment of the study (good quality; fair quality; poor quality) and comments	Good quality- selection bias through lack of untreated late-stage patients in the hospital cohort						

Maranga Et al 2013

Quality criterion	Well covered	Adequately addressed	Poorly addressed	Not addressed	Not reported	N/A	Comments
1. Are the objectives or hypotheses of the study stated?	X						
2. Is the target population defined?	X						
3. Is the sampling frame defined?	X						
4. Is the study population defined?	X						
5. Are the study setting (venues) and/or geographic location stated?	X						
6. Are the dates between which the study was conducted stated or implicit?	X						
7. Are the eligibility criteria stated?	X						
8. Are the issues of 'selection in' to the study mentioned?	X						
9. Are the numbers of participants justified?	X						had a large sample size
10. Was the number of participants at the beginning of the study stated?	X						
11. Were the methods of data collection stated?	X						
12. Was the reliability (repeatability) of measurement methods mentioned?	X						
13. Are the methods of follow-up given?	X						
14. Was the number of participants at each stage/wave specified?	X						
15. Were the reasons for loss to follow-up quantified?	X						
16. Was the missing data items at each wave mentioned?						X	
17. Were missing data accounted for in the analyses?						X	
18. Was the impact of biases estimated quantitatively or quantitatively?	X						
19. Was there any other Discussion of generalizability?	X						
20. Overall assessment of the study (good quality; fair quality; poor quality) and comments	Good quality-data was captured manually						

Moelle et al 2018

Quality criterion	Well covered	Adequately addressed	Poorly addressed	Not addressed	Not reported	N/A	Comments
1. Are the objectives or hypotheses of the study stated?	X						
2. Is the target population defined?	X						
3. Is the sampling frame defined?	X						
4. Is the study population defined?	X						
5. Are the study setting (venues) and/or geographic location stated?	X						
6. Are the dates between which the study was conducted stated or implicit?	X						
7. Are the eligibility criteria stated?	X						
8. Are the issues of 'selection in' to the study mentioned?	X						
9. Are the numbers of participants justified?	X						
10. Was the number of participants at the beginning of the study stated?	X					X	
11. Were the methods of data collection stated?	X						
12. Was the reliability (repeatability) of measurement methods mentioned?	X						
13. Are the methods of follow-up given?						X	
14. Was the number of participants at each stage/wave specified?						X	
15. Were the reasons for loss to follow-up quantified?						X	
16. Was the missing data items at each wave mentioned?		X					
17. Were missing data accounted for in the analyses?		X					No way of getting missing data
18. Was the impact of biases estimated quantitatively or quantitatively?		X					Were not able to fully control for HIV status in the multivariate analysis.
19. Was there any other Discussion of generalizability?	X						
20. Overall assessment of the study (good quality; fair quality; poor quality) and comments	Good quality- unable to ascertain whether patients discontinued RT because of substandard care or whether patients discontinued RT because of fatal progression of the disease						

Owenga, J. A.Nyambedha, E. O. 2018

Quality criterion	Well covered	Adequately addressed	Poorly addressed	Not addressed	Not reported	N/A	Comments
1. Are the objectives or hypotheses of the study stated?	X						
2. Is the target population defined?	X						
3. Is the sampling frame defined?	X						
4. Is the study population defined?	X						
5. Are the study setting (venues) and/or geographic location stated?	X						
6. Are the dates between which the study was conducted stated or implicit?	X						
7. Are the eligibility criteria stated?	X						
8. Are the issues of 'selection in' to the study mentioned?	X						
9. Are the numbers of participants justified?	X						
10. Was the number of participants at the beginning of the study stated?						X	
11. Were the methods of data collection stated?							
12. Was the reliability (repeatability) of measurement methods mentioned?	X						
13. Are the methods of follow-up given?						X	
14. Was the number of participants at each stage/wave specified?						X	
15. Were the reasons for loss to follow-up quantified?						X	
16. Was the missing of data items at each wave mentioned?						X	
17. Were missing data accounted for in the analyses?						X	
18. Was the impact of biases estimated quantitatively or quantitatively?	X						patients could have exaggerated their financial challenges with the hope of receiving some assistance
19. Was there any other Discussion of generalizability?	X						
20. Overall assessment of the study (good quality; fair quality; poor quality) and comments	Good quality- CC patients seeking care at the hospital this patients who were sick at home were left out Such patients may be experiencing worse financial challenges						

Sabulei, C. Maree, J. E. 2019

Quality criterion	Well covered	Adequately addressed	Poorly addressed	Not addressed	Not reported	N/A	Comments
1. Are the objectives or hypotheses of the study stated?	X						
2. Is the target population defined?	X						
3. Is the sampling frame defined?	X						
4. Is the study population defined?	X						
5. Are the study setting (venues) and/or geographic location stated?	X						
6. Are the dates between which the study was conducted stated or implicit?	X						
7. Are the eligibility criteria stated?	X						
8. Are the issues of 'selection in' to the study mentioned?	X						
9. Are the numbers of participants justified?		X					Small sample size
10. Was the number of participants at the beginning of the study stated?						X	
11. Were the methods of data collection stated?	X						
12. Was the reliability (repeatability) of measurement methods mentioned?	X						
13. Are the methods of follow-up given?						X	
14. Was the number of participants at each stage/wave specified?						X	
15. Were the reasons for loss to follow-up quantified?						X	
16. Was the missing data items at each wave mentioned?						X	
17. Were missing data accounted for in the analyses?						X	
18. Was the impact of biases estimated quantitatively or quantitatively?		X					Social desirability
19. Was there any other Discussion of generalizability?	X						
20. Overall assessment of the study (good quality; fair quality; poor quality) and comments	Good quality- Using a cross-sectional design did not allow for the investigation of changes in the same respondents over time						

Tapera Et al2019

Quality criterion	Well covered	Adequately addressed	Poorly addressed	Not addressed	Not reported	N/A	Comments
1. Are the objectives or hypotheses of the study stated?	X						
2. Is the target population defined?	X						
3. Is the sampling frame defined?	X						
4. Is the study population defined?	X						
5. Are the study setting (venues) and/or geographic location stated?	X						
6. Are the dates between which the study was conducted stated or implicit?	X						
7. Are the eligibility criteria stated?	X						
8. Are the issues of 'selection in' to the study mentioned?	X						
9. Are the numbers of participants justified?	X						
10. Was the number of participants at the beginning of the study stated?						X	
11. Were the methods of data collection stated?	X						
12. Was the reliability (repeatability) of measurement methods mentioned?	X						
13. Are the methods of follow-up given?						X	
14. Was the number of participants at each stage/wave specified?						X	
15. Were the reasons for loss to follow-up quantified?						X	
16. Was the missing of data items at each wave mentioned?						X	
17. Were missing data accounted for in the analyses?						X	
18. Was the impact of biases estimated quantitatively or quantitatively?		X					The risk of confounding or interaction effects
19. Was there any other Discussion of generalizability?	X						
20. Overall assessment of the study (good quality; fair quality; poor quality) and comments	Good quality- This research involved descriptive cross sectional surveys whose findings cannot be used to infer causal relationships.						

Vulpe, Et al 2018

Quality criterion	Well covered	Adequately addressed	Poorly addressed	Not addressed	Not reported	N/A	Comments
1. Are the objectives or hypotheses of the study stated?	X						
2. Is the target population defined?	X						
3. Is the sampling frame defined?	X						
4. Is the study population defined?	X						
5. Are the study setting (venues) and/or geographic location stated?	X						
6. Are the dates between which the study was conducted stated or implicit?	X						
7. Are the eligibility criteria stated?	X						
8. Are the issues of 'selection in' to the study mentioned?	X						
9. Are the numbers of participants justified?	X						
10. Was the number of participants at the beginning of the study stated?	X						
11. Were the methods of data collection stated?	X						
12. Was the reliability (repeatability) of measurement methods mentioned?	X						
13. Are the methods of follow-up given?		X					Poor follow-up data
14. Was the number of participants at each stage/wave specified?						X	
15. Were the reasons for loss to follow-up quantified?						X	
16. Was the missing data items at each wave mentioned?		X					Retrospective study
17. Were missing data accounted for in the analyses?	X						
18. Was the impact of biases estimated quantitatively or quantitatively?	X						
19. Was there any other Discussion of generalizability?	X						
20. Overall assessment of the study (good quality; fair quality; poor quality) and comments	Good quality- documentation can be brief and incomplete, charted in an environment with high patient load and Low physician-to-patient ratio.						
