# Supplementary material for: Mapping Evidence on Management of Cervical Cancer in Sub-Saharan Africa: Scoping Review
Source: Int J Environ Res Public Health. 2022 Jul 28;19(15):9207. doi: 10.3390/ijerph19159207 (PMC9367747; doi:10.3390/ijerph19159207)
Supplement: Supplementary file 1 [file ijerph-19-09207-s001.zip › File S4-Summary of Eligible Studies.pdf]

Description of studies for factors associated with high HPV vaccine coverage

Author and publication year	Country	Title	Study design/ study population/ Sample size	Key findings / Conclusions
1. Binagwaho Et al 2012	Rwanda	Achieving high coverage in Rwanda's national human papillomavirus vaccination programme	Cohort<br>98 792<br>Grade 6 girls	Collaboration between private and public institutions in terms of strong ownership.
2. Black, E. Richmond, R. 2019	Rwanda	Prevention of Cervical Cancer in Sub-Saharan Africa: The Advantages and Challenges of HPV Vaccination	Retrospective<br>98 792<br>Grade 6 girls	Financial support from international partners such as GAVI.
3. Delany-Moretlwe Et al 2018	South Africa	An external assessment of the first-dose phase of the vaccination program to evaluate program coverage and vaccine safety and identify factors that influenced implementation	cross-sectional and mixed-methods approach<br>353564<br>Grade 4 girls aged ≥9 years.<br>key informant interviews (N=34	Comprehensive planning, strong commitment by government, early community sensitisation and involving both ministries of health and education.
4. Mabeya Et al 2018	Kenya	Uptake of three doses of HPV vaccine by primary school girls in Eldoret, Kenya	cross-sectional survey<br>3083<br>Girls aged 9 to 14 years from 40 schools.	Empowering teachers to be vaccine champions in disseminating information about HPV vaccine and CC.
5. Msyamboza Et al 2017	Malawi	Implementation of a human papillomavirus vaccination demonstration project in Malawi: successes and challenges	cross-sectional study<br>26,766<br>girls aged 9-13 years	Education of parents and guardians on CC is required to increase coverage of the HPV vaccination
6. Ogembo Et al 2014	Cameroon	Achieving high uptake of human papillomavirus vaccine in Cameroon: lessons learned in overcoming challenges	Case study<br>6851<br>girls aged 9-13 years	Use of mixed method approaches for vaccination<br>Mother–daughter approach in hard-to-reach girls and extending CC screening to mothers to motivate them to get their daughters to vaccination centres.
7. Soi Et al 2018	Mozambique	Human papillomavirus vaccine delivery in Mozambique: identification of implementation performance drivers	Case study<br>Forty key informant interviews<br>Health professionals teachers	Organizational incentives and rewards<br>Being well-informed of the vaccination program and awareness of CC<br>Availability of resources /funds
8. Watson-Jones Et al 2012	Tanzania	Human papillomavirus vaccination in Tanzanian schoolgirls: cluster-randomized trial comparing 2 vaccine-delivery strategies	Case Study<br>cluster-randomized trial<br>3352 and 2180<br>girls in schools randomized to class-based and age-based	Opt-out consent approach whereby parents indicate to teachers that they do not want their daughter to be vaccinated<br>Higher rates with the class-based compared with age-based strategy
9. Watson-Jones Et al 2015	Kenya	Access and Attitudes to HPV Vaccination amongst Hard-To-Reach Populations in Kenya	Stakeholder interviews(n=28) And focus Group(n=14)	Running HPV vaccine programme alongside well known programmes like de-worming programme
10. Vermandere Et al 2014	Kenya	determinants of acceptance and subsequent uptake of the HPV vaccine in a cohort in Eldoret, Kenya	Case study<br>longitudinal study	Adequate education of all stakeholders involved in HPV vaccination

			472 women	A school class-based approach is the most efficient method of vaccinating girls in SSA

Table A3: Description of studies for factors associated with late stage CC presentation at diagnosis

**Author and publication year**	**Country**	**Title**	**Study design/ study population/ Sample size**	**Key findings/ Conclusions**
11. Begoihn et al 2019	Ethiopia	Cervical cancer in Ethiopia - predictors of advanced stage and prolonged time to diagnosis	retrospective cohort study<br>1575<br>CC patients	longer patient intervals high age and rural residence<br>socioeconomic factors like low education and illiteracy<br>Fear of finding cancer<br>financial and logistic<br>lack of awareness
12. Dunyo, P. Effah, K. Udofia, E. A. 2018	Ghana	Factors associated with late presentation of CC cases at a district hospital	retrospective study<br>157<br>CC patients	Previously unscreened cases biological behaviour of the tumour<br>low socioeconomic status<br>HIV infection, non-use of condoms, high parity
13. Mlange Et al 2016	Tanzania	Patient and disease characteristics associated with late tumour stage at presentation of CC	cross-sectional study<br>202 CC patients	attending to traditional and spiritual healers from rural area<br>being peasant farmers Lack of formal education<br>Lack of specificity of CC symptoms and inadequate facilities for diagnosis<br>misinterpretations of CC symptoms and subsequent diagnosis of non-CC<br>Lack of population-based CC screening
14. Mushosho Et al 2011	Zimbabwe	Presentation patterns of invasive cancer of the cervix:	Retrospective study<br>675<br>CC patients	low socio-economic status not employed<br>high parity
15. Mwaka et al 2016	Uganda	Social, demographic and healthcare factors associated with stage at diagnosis of CC	Cross-sectional<br>149 CC patients	no formal education<br>not perceiving their CC symptoms as serious or due to CC<br>Long distances from diagnostic facilities Delayed recognition or referral by primary healthcare professionals<br>Unmarried women
16. Nassali Et al 2018	Botswana	Predictors of Locally Advanced Disease at Presentation and Clinical Outcomes Among Cervical Cancer Patients Admitted at a Tertiary Hospital in Botswana	Retrospective<br>149 CC patients	Unmarried<br>lack of CC screening<br>older age<br>low social<br>economic status<br>Lack of cytology laboratory and arrangements to communicate results to screened women and facility for confirming diagnosis.
17. Ndlovu, N. Kambarami, R. 2003	Zimbabwe	Factors associated with tumour stage at presentation in invasive CC	A cross sectional study.<br>100 CC patients	Poorly differentiated tumours<br>no prior screening<br>residing in the rural areas<br>unemployed and peasant farmers<br>illiteracy of the patient non-functioning or inadequate screening elderly, unmarried and women without medical insurance

18. Rudd et al 2017	Malawi	Cervical cancer in southern Malawi: A prospective analysis of presentation, management, and outcomes	case series prospective 300 CC patients	Prolonged antibiotic use before referral Treatment from traditional healers and herbalist resulted in delay in seeking health care delay in biopsy results overbooked clinics.
19. van Schalkwyk, S. L. Maree, J. E. Wright, S. C. 2008	South Africa	CC: the route from signs and symptoms to treatment	qualitative study phenomenological 15 women with advanced CC	Lack of knowledge, awareness among health care professionals resulting in a low suspicion of CC and misdiagnosis<br>Poverty, long distances to clinics, low educational levels, lack of transportation, and social isolation.<br>poor referral system of the health care system<br>lack of knowledge about the disease Cultural beliefs that abnormal vaginal bleeding was caused by witchcraft and that the body is cleansing itself<br>traditional healers<br>mismanagement by health care workers
20. Wamburu Et al 2016	Kenya	Association between stage at diagnosis and knowledge on cervical cancer among patients in a Kenyan tertiary hospital	A cross-sectional survey 361 CC patients	not aware of causative link between CC and HPV lack of specialised health practitioners poor implementation of CC screening programs absence of frequent gynaecological examinations and lack of awareness on importance of regular gynaecological examinations<br>patient delay<br>practitioner delay<br>system delay

Table A4: Description of studies for barriers to CC screening uptake

Author and publication year	Country	Title	Study design/ study population/ Sample size	Key findings/ Conclusions
21. Bateman et al 2019	Tanzania	Barriers and Facilitators to Cervical Cancer Screening, Diagnosis, Follow-Up Care and Treatment: Perspectives of Human Immunodeficiency Virus-Positive Women and Health Care Practitioners	6 focus groups<br>61 Participants<br>HIV positive<br>Women<br>and<br>Clinicians	fear and stigma surrounding CC<br>lack of knowledge and access to screening services<br>Socio-cultural beliefs that diagnosis leads to death/ if you know early you die early<br>fear that screening could lead to discovery of CC<br>lack of training and motivation as cc screening was treated as tusk shifting
22. Binka, C. Nyarko, S. H. 2019	Ghana	Barriers to the Uptake of Cervical Cancer Screening and Treatment among Rural Women in Ghana	Focus groups<br>In-depth interviews<br>55<br>CC patients<br>Women who have not screened(30-65yrs)	low level of and cost of screening<br>Negative health personnel attitude lack of privacy<br>Socio cultural belief about the aetiology of CC<br>lack of funding at policy level<br>belief in traditional medicine normative gender relations and need for approval of partners to undergo screening<br>normative gender relations and need for approval of partners to undergo screening<br>Lack of policies on management of CC or poor implementation of the existing policies<br>lack of government subsidy on CC screening<br>Unavailability and inaccessibility of screening facilities

				Myths and misconceptions a woman's ovaries and uterus are removed during screening<br>fear of pain associated with CC screening<br>low perceived CC risk<br>shortage of health professionals to routinely do CC education and screening, and competing priorities for provider and the women for time<br>preference for divine intervention instead of screening<br>Hostile attitude of the health
23. Bukirwa<br>et al<br>2015	Uganda	Motivations and barriers to CC screening among HIV infected women	cross-sectional qualitative study<br>HIV infected women<br><br>health care providers<br><br><br>18 in-depth interviews<br><br>6 key informant interviews	Long waiting time<br>Inadequate knowledge about CC and its prevention as well as long distance to the screening sites<br>Fear of stress from an additional diagnosis<br>Competing health priorities as well low prioritisation of CC screening<br>Lack of a proper follow-up procedures<br>Low staff numbers as well as negative staff attitude towards CC screening<br>need for partner's approval
24. Ilevbare, O. E.<br>Adegoke, A. A.<br>Adelowo, C. M.<br>2020	Nigeria	barriers to CC screening among Nigerian women	cross-sectional study<br>852	The beliefs that CC is caused by breach of social taboos negative attitude towards CC screening as a prevention methods cost of screening , pain of the procedure and being attended by male medical stuff
25. Isa<br>Modibbo, F.<br>Dareng, E.<br>Bamisaye, P.<br>2016	Nigeria	Barriers to CC screening, focusing on religious and cultural factors, in order to inform group-specific interventions that may improve uptake of CC screening programmes.	Four focus group discussions<br>27 Christian and 22 Muslim women over the age of 18, with no diagnosis of cancer	Do not feel at risk of cervical cancer<br>lack of awareness<br>discrimination at hospitals<br>The belief that it is better to be ignorant of the disease than to go in search of it<br>Low levels of trust in the healthcare system.<br>violations of religious and cultural obligations of modesty during screening procedures
26. Kivuti-Bitok<br>et al<br>2013	Kenya	An exploration of opportunities and challenges facing CC managers in Kenya	qualitative study using interview guide<br>21nurses and 12doctors.	limited training among health care providers<br>Culturally unacceptable for male to see female private parts.<br>Lack of health care insurance. Healthcare workers view CC screening as a burden.<br>Health-care systems are donor funded and focus on specific diseases like TB ,HIV, malaria and maternal health<br>Women's perception of low threat of CC<br>Large workload compromised quality of care given to patient seeking screening services.

				Suspicious of infections like HIV from the use of speculum which are not disposable
27. Lee<br>Et al<br>2020	Malawi	Health systems challenges in CC prevention program in Malawi	A rapid ethnographic approach<br>11 women and 7 men	Affordability and donor-driven systems<br>Social inequalities in rural areas , poor road conditions lack of public transport<br>Health care practice is based on management of acute infectious diseases<br>No standards and guidelines for CC screening<br>No service provider allocated specifically for screening CC.<br>Negative perception of paying for health services<br>Beliefs and misconceptions about causes of CC
28. Maseko, F. C.<br>Chirwa, M. L.<br>Muula, A. S.<br>2015	Malawi	Health systems challenges in cervical cancer prevention program in Malawi	exploratory cross-sectional<br>41 service providers from 21 health facilities and 9 district coordinators	Insufficient funding for implementation of screening programs<br>lack of equipment and supplies<br>lack of supportive supervision<br>use of male service providers<br>Services are not offered everyday<br>Widespread poverty<br>Gross shortage of staff<br>long distances to health facilities,
29. Munthali, A. C.<br>Ngwira, B. M.<br>Taulo, F.<br>2015	Malawi	Exploring barriers to the delivery of CC screening and early treatment services in Malawi: some views from service providers	in-depth interviews<br>13 district coordinators and 40 service providers	Negative individual perceptions- having no signs and symptoms not being at risk, lack of time and fear of the outcomes<br>Not aware of CC screening services<br>health facility challenges<br>distance, costs , long waiting times<br>lack of partner participation
30. Ndejjo<br>Et al<br>2016	Uganda	Uptake of CC Screening and Associated Factors among Women in Rural Uganda	A Cross Sectional Study<br>900<br>females aged between 25 and 49 years	domestic gender power relations alternative sources of reproductive health<br>unfriendly health care services<br>illiteracy<br>belief in not being at risk<br>having many contending issues<br>nonchalant attitude to personal health<br>financial constraints
31. Shiferaw, S.<br>Addissie, A.<br>2018	Ethiopia	Knowledge about CC and barriers toward cervical cancer screening among HIV-positive women attending public health centres	A Cross Sectional Study<br>581<br>HIV-positive women aged 21-64 years	opportunistic CC screening program<br>feeling healthy<br>lack of health provider recommendations<br>absence of health education<br>Lack of knowledge on CC and CC screening
32. Were, E.<br>Nyaberi, Z.<br>Buziba, N.	Kenya	Perceptions of risk and barriers to CC screening at Moi Teaching and Referral Hospital (MTRH), Eldoret, Kenya	Cross-sectional questionnaire survey<br>219<br>non-pregnant women	low priority accorded to women's health<br>fear of genital exam<br>lack of correct perception on own risk of developing CC

2011				lack of awareness on screening service<br>lack of support from husbands
33. Williams<br>Et al<br>2013	Ghana	Assessment of psychological barriers to CC screening among women in Kumasi, Ghana using a mixed methods approach	Semi-structured interviews were conducted with 49 Ghanaian women with cancer and 171 Ghanaian women who did not have cancer.	myths and misconceptions about CC screening<br>cultural taboos regarding the gender of healthcare providers<br>women's attitudes, misconceptions, and beliefs about CC<br>stigmatisation of women with CC<br>lack of spousal support for CC screening<br>lack of knowledge about CC<br>lack of social support
34. Getachew, S.<br>Getachew, E.<br>2019	Ethiopia	Drivers of cervical cancer screening uptake in Ibadan, Nigeria	cross-sectional study<br>520<br>Women visiting reproductive clinics<br>Four focus group discussions<br>37 women were involved	lack of health worker request<br>high costs of screening<br>lack of a screening service in their living area<br>fear of positive outcomes<br>religious and cultural beliefs<br>financial constraints<br>lack of health personnel at screening centres<br>Availability, accessibility and affordability<br>lack of knowledge on screening

Table A5: Description of studies for status chemotherapy

**Author and publication year**	**Country**	**Title**	**Study design/ study population/ Sample size**	**Key findings/ Conclusions**
35. Einstein<br>Et al<br>2019	South Africa<br>Zimbabwe		A phase II study<br>38<br>Eligible participants had HIV infection and untreated, histologically-confirmed, invasive carcinoma of the uterine cervix	HIV-infected women with CC can complete chemo radiotherapy with the same cisplatin dose which is used in HIV-negative women with almost the same tolerability and good Anti-Retrovirus adherence while on CC treatment.<br>to avoid zidovudine and tenofovir because of overlapping neurological, hematologic, and renal toxicities<br>The most common adverse event was decreased lymphocyte count that affected all treated patients<br>adverse events in treated participants included:<br>Diarrhoea<br>Vomiting<br>Chronic Kidney Disease<br>Syncope<br>Hypermagnesemia<br>Hypokalemia<br>Hypomagnesemia<br>Hypertension

				Non-hematologic serious adverse events were similar to those observed in women with without HIV infection
36. Martei et al 2018	Botswana	Availability of WHO Essential Medicines for Cancer Treatment in Botswana	Document Review<br>18 drugs<br>Interviews with key informants (from pharmacy and oncology) were used to collect data on the Botswana NEML and the drug supply chain in the public sector.	Inefficiency in procurement in the public sector.<br>Most chemotherapy drugs were out of at the end of the due to insuffient budget allocation<br>Funding was inadequate to cover pharmaceuticals needed for CC treatment of CC diagnosed annually.<br>Lack of standardised treatment protocols.<br>Patients experienced suboptimal therapy due to delays in therapy, missed doses, substitution with less efficacious as well as substitution with more costly chemotherapy drugs<br>lack of accurate chemotherapy drugs forecasting<br>At least 40% of essential drugs were out of stock<br>Shortages were related to weak infrastructure for the procurement as well as distribution<br>Alignment with WHO National Essential Medicine List for SSA was 34.1% from HICs 73.2%
37. McArdle, O. Kigula-Mugambe, J. B. 2007	Uganda	Contraindications to cisplatin based chemo radiotherapy in the treatment of CC in Sub-Saharan Africa	prospective study<br>314<br>CC patients	Common exclusion criteria for chemotherapy were hydronephrosis and anaemia.<br>HIV positive patients were more likely to meet multiple exclusion criteria<br>More than sixty percent of women seeking treatment are not suitable for chemo radiotherapy at the time Of presentation.<br>failure to establish eligibility for chemo radiotherapy was due to economic, geographic, social and psychological factors<br>concomitant chemo radiotherapy produce an overall survival advantage of 10–16% in the treatment of CC
38. Simonds Et al 2012	South Africa	Completion of and early response to chemo radiation among (HIV)-positive and HIV-negative patients with locally advanced cervical carcinoma in South Africa		a poor response was associated with late stage presentation<br>Renal dysfunction was the common reason for not completing chemotherapy<br>Chemotherapy component is the difficult aspect of chemo radiation for HIV-positive patients to complete.<br>Patients who failed to complete chemotherapy had lower CD4 counts than those who completed it.

Table14: Descrption of studies for status of radiotherapy

**Author and publication year**	**Country**	**Title**	**Study design/ study population/ Sample size**	**Key findings/ Conclusions**
39.Akinlade et al 2015	Nigeria	Radiation therapy interruption in a poor resource setting: causes and management	retrospective study<br>500<br>cervical carcinoma	most patients experienced treatment interruption in the Course of their management due: to machine Breakdown

			patients	financial challenges<br>ill health due to disease and side effects of treatment
40. Du Toit, G. C.<br>Kidd, M.<br><br>2015	South Africa.	Prospective quality of life study of South African women undergoing treatment for advanced-stage CC	Prospective study<br>219<br>CC patients	The effect of chemo radiation therapy on survival decreased with increasing tumour stage<br>5-yeararsurvival<br>Benefit of 29% in all stages of cervical cancer for chemo radiation therapy compared with radiation therapy.<br>Chemo radiation therapy improved quality of life more than radiation therapy in certain domains.
41. Einck<br>et al<br>2014	Senegal	Implementation of a high-dose-rate brachytherapy program for carcinoma of the cervix in Senegal	Case study<br>Experiment<br>CC patients	there was a lack of necessary infrastructure and equipment<br>a large up-front training requirement for physics and radiation oncology staff<br>5-fluorouracil and cisplatin as chemotherapy<br>A high-dose brachytherapy implementation is possible in developing countries with fixed geometry<br>Applicators with a library of plans
42. Kamau, R. K.<br>Osoti, A. O.<br>Njuguna, E. M.<br>2007	Kenya	Effect of diagnosis and treatment of inoperable CC on quality of life among women receiving radiotherapy at Kenyatta National Hospital	Cross-sectional descriptive study<br>152<br>Women undergoing radiotherapy	disruptions in all domains of QOL<br>perception that family members and friends had withdrawn social support<br>fall in the overall living standards<br>sexual domain, as a patients reported marital discordance<br>personality domain-decreased self-esteem<br>Cognitive functions
43. Kantelhardt<br>Et al<br>2014	Ethiopia	CC in Ethiopia: survival of 1,059 patients who received oncologic therapy	prospective cohort study<br><br>1059<br>CC patients	majority of patients presented with late stage CC<br>Palliative radiotherapy was administered to almost half of the patients because of the lack of finances<br>waiting time was 2.3 months between first registration at the Radiotherapy Centre and the first appointment with the radiation oncologist<br>The median survival time was 21.5 months<br>900 patients who registered never received any therapy<br>An important piece of baseline information is change in FIGO stage between pathological diagnosis and the start of radiotherapy, during which time a considerable number of patients died while waiting for treatment.<br>Twenty-eight patients experienced recurrence.<br>high doses showed better outcome<br>most patients unable to pay for therapy<br>Radiotherapy did not include the application of brachytherapy.
44. Maranga<br>Et al<br>2013	Kenya	Analysis of factors contributing to the low survival of CC patients undergoing radiotherapy in Kenya	prospective cohort study<br>355<br>patients with histological confirmed ICC	80.5% of patients presented with advanced stage<br>6.7% of patients receiving optimal combined EBRT, brachytherapy and adjuvant chemotherapy<br>Kaplan Meier survival curves projected two year survival at <20%<br>radiological investigations like MRI, CT-Scan and ultra-sound scans were not routinely done because of cost-implications<br>brachytherapy equipment was non-functional<br>Commonly used chemotherapy drugs were cisplatin and 5-fluorouracil

				15% had actually ever had a pap smear<br>the mean time to death after the onset of treatment was 15.1 months<br>No deaths occurred directly due to acute treatment toxicity
45. Moelle et al<br><br>2018	Ethiopia	CC in Ethiopia: The Effect of Adherence to Radiotherapy on Survival	Retrospective<br>788<br>patients with CC who received RT	Reasons for discontinuation were toxicities, economic background, and RT machine breakdown<br>Discontinuation of planned radiotherapy reduced survival for all stages treated<br>Most patients originated from rural Ethiopia (58%)<br>Chemotherapy was administered in only 12 cases.<br>Side effects of radiotherapy included radiation proctitis, Subcutaneous fibrosis of the suprapubic tissue and vaginal strictures incontinent because of vesicovaginal fistula<br>Radiation dermatitis and diarrhoea<br>patients had lower survival probabilities in the case of discontinuation<br>only one RT machine existed in the country<br>Parity ranged from 0 to 17 children<br>morphine injections were given to only 14% of all patients<br>27% did not receive any analgesic<br>73 RT units missing in Ethiopia
46. Owenga, J. A. Nyambedha, E. O.<br>2018	Kenya	Perception of Cervical Cancer Patients on their Financial Challenges in Western Kenya	A cross-sectional study<br>334 CC patient	The financial challenges included costs of medication cost of travel cost of diagnostic tests cost of cloths and wigs cost of home, hygienic needs and child care<br>Most (91%) of the CC patients did not have insurance cover<br>Radiotherapy machine was available but was not operational<br>The facility did not have an oncologist, there were three palliative care nurses, a pathologist (mainly conducting biopsies) and gynecologists who staged cervical cancer and conducted surgery where appropriate<br>For other associated diagnostic and routine laboratory tests the patients would be referred to the private facilities<br>67% not screened for CC<br>22% were HIV negative<br>9% were formarly employed<br>10% received chemotherapy<br>3% received chemo radiation<br>most of the patients depended on their relatives, church members and their children for financial assistance most of whom did not have any formal employment or steady source of income<br>lack of decentralized diagnostic and treatment facilities<br>For palliation, chemotherapy including cisplatin, paclitaxel and/or bevacizumab
47. Sabulei, C. Maree, J. E.<br>2019	South Africa	An exploration into the quality of life of women treated for CC	A cross-sectional design<br>153<br>CC patients	Insomnia and urinary frequency were cumbersome problems and remained so even after treatment<br>Financial difficulties had the highest mean score<br>general problems, consisting of fatigue, pain, insomnia, dyspnoea, swelling of the feet and tingling of the hands and feet<br>gastrointestinal symptoms, nausea, vomiting, diarrhoea and appetite loss

				urological data included difficulty in emptying the bladder , urinary frequency, burning sensation when urinating and urine leakage<br>Vaginal discharge and vaginal bleeding<br>sexual activity ,enjoyment and vaginal functioning declined after treatment<br>screening coverage is as low as 13%resulting in most women presenting with advanced disease<br>brachytherapy was a negative, humiliating experience causing pain and fear
48. Tapera<br>Et al<br>2019	Zimbabwe		Survey<br>Indepth intervies<br>Focus group<br>134 CC<br>Patients<br>78 health workers<br>20 in-depth interviews<br>6 focus group	on out-of-pocket funding for treatment services<br>lack of back-up for major equipment<br>: high costs of treatment and care<br>treating centres located mostly in urban areas<br>staging and monitoring investigations which are provided in private facilities as public health facilities do not have sufficient capacity to offer them<br>Treating health facilities do not provide chemotherapy drugs and they refer patients to private pharmacies
49. Vulpe,<br>Et al<br>2018	Ghana	External Beam Radiation Therapy and Brachytherapy for Cervical Cancer: The Experience of the National Centre for Radiotherapy in Accra, Ghana	retrospective<br>250<br>CC patients	Cobalt 60 external beam radiation therapy was followed by 2 low-dose-rate brachytherapy insertions. Concurrent weekly cisplatin was recommended<br>treatment time was 73 days<br>The most commonly reported late side effect was vaginal stenosis and shortening, proctitis/rectal bleeding , hematuria , subcutaneous fibrosis rectovaginal , vesicovaginal fistulas<br>Reasons for interruptions<br>included severe anemia or neutropenia , GI toxicity machine breakdown and insufficient funds<br>reasons for incomplete chemotherapy<br>included GI toxicity, myelosuppression, insufficient funds and nephrotoxicity<br>23 of 52 African countries with RT centres only 20 with brachytherapy facilities
